# Development of Diagnostic Capabilities for Complications of Bacterial Infection in Diabetic Patients

**DOI:** 10.1900/RDS.2022.18.135

**Published:** 2022-06-30

**Authors:** Samiah Hamad S Al-Mijalli, Ashwag Y Shami, Rasha A Al-Salem, Nawaf M Alnafisi

**Affiliations:** 1 Department of Biology, College of Sciences, Princess Nourah bint Abdulrahman University, P.O.Box 84428, Riyadh 11671, Saudi Arabia,; 2 College of Medicine, King Saud bin Abdulaziz University for Health Sciences, Riyadh, Saudi Arabia.

**Keywords:** UTI, bacteremia in diabetics, antimicrobial susceptibility, antibiotic resistance, multidrug-resistant organisms, bacterial infection

## Abstract

**OBJECTIVE:**

Our objective was to assess the pattern of urine infections, the most common pathogen, and their susceptibility pattern to antibiotics among Saudi diabetic patients.

**METHODS:**

We performed a year-long cross-sectional study from January 2018 to January 2019 at KAAU Hospital in Riyadh, KSA. We cultured the urine specimens obtained from diabetic patients based on optimal aerobic and anaerobic microbiological methods. By adopting standard microbiological methods, we identified the bacterial isolates. We also followed the guidelines of the Clinical and Laborator y Standards Institute (CLSI) to do antibiotic susceptibility testing.

**RESULTS:**

A total of 100 isolates were evaluated, and a total of 22 organisms were isolated. The majority were multidrug-resistant organisms. *Streptococcus haemolyticus* was the most frequent organism and rated (15%). It was followed by *Staphylococcus hominis* (11%), *Pseudomonas aeruginosa* (9%), *Enterococcus faecalis* (9%), *Enterococcus fiseum* (7%), *Escherichia coli* (7%), *Staphylococcus aureus* (7%), *Staphylococcus lantus* (5%) and *Klebsiella pneumoniae* (5%). We also found multi-microbial infections. Most of the organisms were susceptible to tigecycline, gentamycin, and nitrofurantoin, rating (88%), (84%) and (78%), respectively.

**CONCLUSIONS:**

Our study revealed that a wide range of pathogens affects the diabetes patients. *Staphylococcus haemolyticus* is the most prevalent pathogen. We observed considerable antimicrobial resistance. Tigecycline had a wide sensitivity spectrum and was effective against most of the bacteria. Thus, it can be used as an empirical antibiotic.

## 1 Introduction

Diabetes mellitus (DM) is a universal and pervasive health problem. In some countries, including Saudi Arabia, DM achieves epidemic proportions [1]. In 2000, it affected about 171 million people worldwide. Eleven years later, this number grew to 366 million. Furthermore, some authorities estimate that DM may affect more than 552 million by 2030 [2].

According to the World Health Organization (WHO) – Diabetes Country Profiles, 2016, the prevalence of DM is 14.7% in males and 13.8% in females [3]. The International Diabetes Federation (IDF) argues that there are 451 million persons worldwide (aged 18-99) with DM. There were 415 million people in 2017. It is estimated that there may be 693 million with DM by 2045.

DM affects multiple aspects of patients’ lives, including quality of life and employment. It may result in premature death. It is ranked as the fifth cause of death in developed countries [4]. Therefore, patients with DM are increasingly at risk to asymptomatic bacteriuria and pyuria, cystitis, as well as serious upper urinary tract infections (UTIs) [5,6].

Once the urine glucose level is elevated and the host immune factors are impaired, a person is predisposed to infection. A patient may encounter neutrophil dysfunction due to hyperglycemia that elevates intracellular calcium levels, interfering with actin. As a result, a patient has diapedesis and phagocytosis.

Although variations occur, most common relevant UTI-related bacteria in DM are *Escherichia coli (E. coli), Proteus* (multiple species), *Klebsiella* (multiple species), *Pseudomonas aeruginosa, Enterococcus faecalis, Staphylococcus aureus*, and coagulase-negative staphylococci [7-9]. Patients with DM are prone to mortality more than those without DM [10]. They are also at a greater risk of UTIs and complications, especially those with type 2 DM. One survey concluded that UTIs are the most common microbial infections worldwide [11] and the most prevalent among DM patients [12]. Moreover, UTIs explain a massive proportion of antibacterial drug consumption and have considerable fiscal impact [13].

UTIs indicate the presence of microbial pathogens inside the urinary tract. A UTI is commonly categorized according to the location of infection – bladder (cystitis), kidney (pyelonephritis), or urine (bacteriuria). UTIs may be either asymptomatic or symptomatic. As an infection, a UTI prevails among patients, regardless of the age group [14].

*E. coli* often is ranked first in terms of causing UTIs. *Klebsiella* and *Proteus* species are ranked second and are often related to stone disease. Moreover, Gram-positive bacteria, eg, *Enterococcus* and *Staphylococcus*, increase [15,16].

Despite abundant research, alterations in the phagocyte function, as well as augmented, attenuated, or unchanged cytokine responses to infection in relation to DM are not well defined. Thus, the immunological foundation of DM-intracellular bacterial infections requires further examination [17-21].

Previous studies have shown reduced functioning of diabetic polymorphonuclear cells and diabetic monocytes/macrophages, including chemotaxis, phagocytosis, killing of bacteria, as compared to cells of control individuals. Moreover, some microorganisms pose severe threats in an environment with increased glucose content. Evidence suggests that the more microorganisms adhere to DM cells, the more prevalent infections among DM patients become. In addition, serum TNF-α level in type 1 DM patients has increased considerably, regardless of age, disease duration, and ethnicity groups [22,23].

Bacteremia is a serious health concern. It indicates that viable bacteria are in the bloodstream, as shown by blood cultures. It causes mortality in up to 20% of cases. Therefore, in-depth research-based knowledge is an important objective for improving patient prognosis.

Hyperglycemia is damaging. It causes dysfunction and failure of various organs. It mainly affects the eyes, kidneys, nerves, heart, and blood vessels. Hyperglycemia leads to defective phagocytosis and reduced immunity [24].

## 2 Methods and materials

We conducted a year-long prospective study on Saudi type 2 DM patients from January 2018 to January 2019 at KAAU Hospital in Riyadh, Kingdom of Saudi Arabia. We also studied the media for bacterial growth. In short, we identified the bacteria isolates from urine by conventional biochemical testing. A total of 100 isolates were analyzed for c/s.

All colonies were grown on agar of blood culture bottles, subjected to direct Gram staining, and subcultured onto nutrient agar, blood agar, mannitol salt agar, MacConkey agar and Sabouraud’s dextrose agar. They were incubated at 37°C under aerobic and anaerobic conditions for 24-hours.

The identification of aerobic and facultative anaerobes was done according to standard microbiological methods and antibiotic sensitivity of isolated aerobic and facultative anaerobes [25,26]. The antimicrobial susceptibility was defined based on the disk diffusion method. It was interpreted using the CLSI.

We collected 100 samples during the study period. To isolate the microbial agents of UTIs, we cultured urine specimens using blood agar and MacConky agar media. Then, we incubated the media at 37°C aerobically for 24-hours. We also studied the media for bacterial growth. In short, we identified bacteria isolates from urine by conventional biochemical testing.

### 2.1 Antibiotic susceptibility testing

We conducted antimicrobial susceptibility tests on Mueller-Hinton agar utilizing disk diffusion (Kirby Bauer’s) method following the CLSI guidelines and using the following antimicrobial agents – Amikacin, Gentamicin, Ciprofloxacin, Ertapenem, Nitrofurantoin, Imipenem, Meropenem, Trimethoprim/ Sulfamethoxazole, Tigecycline, Piperacillin/ Tazobactam, Levofloxacin, Colistin, Cephalothin, Cefuroxime, Ceftriaxone, Ceftazidime, Cefoxitin, Cefepime, Aztreonam, Ampicillin and Amoxicillin rating (30 μg), (10 μg), (5 μg), (30 μg), (300 μg), (30 μg), (30 μg), (25 μg), (30 μg), (30 μg), (30 μg), (10 μg), (30 μg), (30 μg), (30 μg), (30 μg), (30 μg), (10 μg), (30 μg), respectively for all bacterial isolates.

### 2.2 Statistical analysis

We used the Statistical Package for Social Sciences (SPSS), version 21.0, for data analysis. We presented data in the form of frequencies and percentages.

## 3 Results

### 3.1 Demographic characteristics

Table 1 illustrates the demographic characteristics of the population. A total of 100 patients with type 2 DM participated. There were 47% males 53 % females, with a mean age of 37+14 years, and duration of DM 7.2+3.5 years duration of UTIs. Comorbidities among patients included hypertension (22%), cardiovascular disease (14%), and nephropathy (9%).

**Table 1.** Sample demographics

Variables	NO %
Age (Mean + SD)	37+14 years
Gender:	47%
Male	53 %
Female	
Duration of Diabetes	
Duration of UTI	
Duration of diabetes mellitus (DM)	7.2+3.5 years
Hypertension	22%
Cardiovascular disease	14%

### 3.2 Pathogens isolated from type 2 diabetes mellitus patients

A total of 100 isolates were evaluated, and 22 organisms were isolated. The majority were multidrug-resistant organisms. *Streptococcus haemolyticus* was the most frequent organism in 15% of cases. It was followed by *Staphylococcus hominis* (11%), *Pseudomonas aeruginosa* (9%), *Enterococcus faecalis* (9%), *Enterococcus fiseum* (7%), *E.coli* (7%), *Staphylococcus aureus* (7%), *Staphylococcus lantus* (5%), and *Klebsiella pneumoniae* (5%). We also found multi-microbial infections, as Table 1 shows.

### 3.3 Microbial susceptibility

The salient antibiotics tested were Roxithromycin, Clindamycin, Aztreonam, Imipenem, Meropenem, Amikacin, Gentamicin, Ciprofloxacin, Norfloxacin, Ofloxacin, Doxycycline, Minocycline, Tigecycline, Nitrofurantoin, Trimethoprim, Vancomycin, Tetracycline, Erythro, Azithromycin, Levofloxacin, Clarithromycin, Amoxicillin, Flomoxef, Benzylpenicillin, Ampicillin, Cefixime, Cefetoxime, Ceftriaxone and Methicillin.

**Figure 1.**
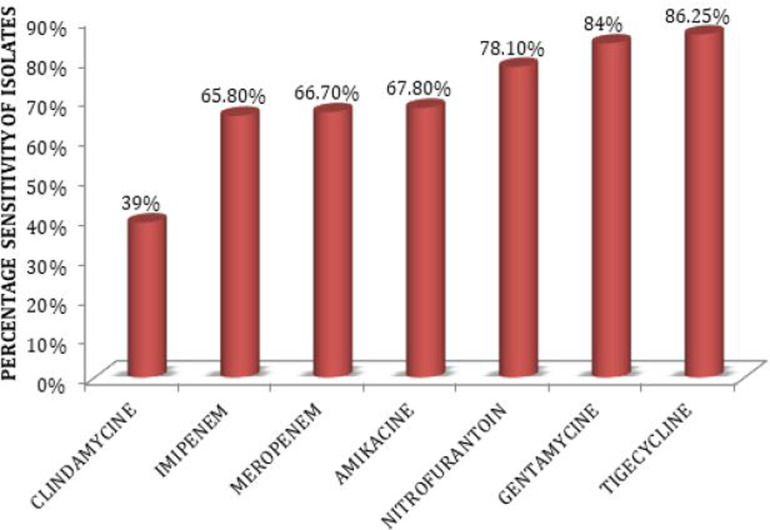
Antimicrobial sensitivity of bacterial isolates.

With respect to the pathogens we found, the most effective antibiotic was Tigecycline, because 86.25% of total isolates were sensitive to it, whereas just 12% resisted it. It was followed by Gentamycin, because 84% of isolates showed sensitivity to it. Nitrofurantoin was ranked third, to which 78% of isolates were sensitive, as Figure 1 illustrates.

### 3.4 Resistance to salient antibiotics

Antimicrobial sensitivity tests were performed on 100 bacterial isolates from Saudi patients with DM. Tigecycline was the most active agent on the 100 isolates. About 61% of isolates were resistant to Clindamycin, 34.2% to Emipem, 33.3% to Meropenem, 32.2% to Amikacin, 16.04% to Gentamicin, 12% to Tigecycline and 21.9% to Nitrofurantoin.

### 3.5 Resistance pattern of salient pathogens

#### 3.5.1 Staphylococcus haemolyticus

**Figure 2.**
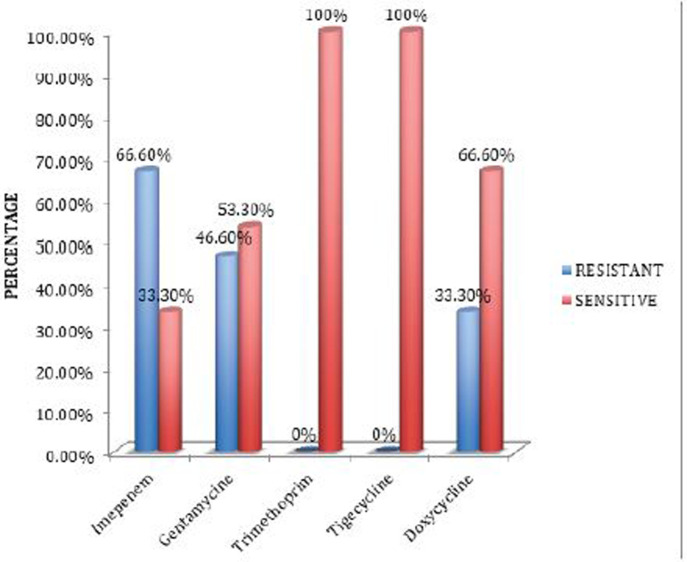
Antimicrobial resistance of *Staphylococcus haemolyticus*.

*Staphylococcus haemolyticus* was the most frequent pathogen in our study. It was most sensitive to Tigecycline and Trimethoprim followed by Doxycycline and Gentamicin. *Streptococcus aeruginosa* was resistant to Trimethoprim, Tigecycline and Doxycycline, but it was sensitive to Gentamicin and Imipenem (Figure 2).

#### 3.5.2 Pseudomonas aeruginosa

*Pseudomonas aeruginosa* was sensitive to Imipenem and Gentamicin, and resistant to Tigecycline, Trimethoprim tigecycline and Doxycycline.

#### 3.5.3 Klebsiella pneumoniae

*Klebsiella pneumoniae* was sensitive to Tigecycline, Trimethoprim, Nitrofurantoin, Doxycycline and Gentamicin.

#### 3.5.4 Enterococcus faecalis

*Enterococcus faecalis* was sensitive to Ciprofloxacin, Nitrofurantoin and Vancomycin. It was resistant to Tetracycline, Trimethoprim and Erythromycin.

## 4 Discussion

Identifying the local or regional causes of UTIs and antimicrobial resistance can help define empirical therapy. We observed that the frequency and features of pathogens vary according to time and region.

Because of the prevalence of these infections, proper treatment plays a considerable role in a patient’s health, development of antibiotic resistance and healthcare costs.

Resistance to commonly advised antibiotics is a global problem. Our study showed that *Streptococcus haemolyticus* was the most frequent organism, present in 15% of UTIs associated with DM. However, other studies have reported that *E. coli* is the most frequent uro-pathogen [27,28].

The Gram-positive, β-hemolytic, chain-forming bacterium *Streptococcus haemolyticus* causes serious infection among neonates, pregnant women, and the elderly with chronic medical illness [29]. However, it was most sensitive to Tigecycline and Trimethoprim, followed by Doxycycline and Gentamicin.

The high prevalence of antimicrobial drug resistance in bacteria has become a major health concern. Therefore, new antimicrobial agents, including Tigecycline, should be tested against the widest possible range of these resistant organisms globally [30]. We illustrated in our study that Tigecycline is the best antimicrobial agent to which 86.25% of bacterial isolates are sensitive. Our findings approach those of another study [31] where the tested Gram-positive organisms were 100% susceptible to the therapeutic effects of Tigecycline.

*Staphylococcus hominis* was the second most frequent resistant bacterium and rated (11%), followed by *Pseudomonas aeruginosa* (9%), *Enterococcus faecalis* (9%), *Enterococcus fiseum* (7%), *E.coli* (7%), *Staphylococcus aureus* (7%), *Staphylococcus lantus* (5%) and *Klebsiella pneumoniae* (5%). We also found multi-microbial infections.

Regarding the pathogens we found in our study, the most effective antibiotic was Tigecycline because 86.25% of total isolates were sensitive to it, and just 12% resisted it. It was followed by Gentamycin because 84% isolates showed sensitivity to it. Nitrofurantoin was ranked third to which 78% isolates were sensitive, as Table 2 shows.

**Table 2.** Spectrum of salient uro-pathogens in Saudi diabetic patients with infection

Pathogens	Number %
*Streptococcus haemolyticus*	15 (15%)
*Pseudomomasaerugimosa*	9 (9%)
*Enterococcus faecalis*	9 (9%)
*Staphylococcushoiinis*	11 (11%)
*Klelsiella pneumoniae*	5 (5%)
*Staphylococcus epidermidis*	4 (4%)
*E.coli*	7 (7%)
*Staphylococcus aureus*	7 (7%)
*Aeromomas hydrophila*	2 (2%)
*Enterococcus faecium*	7 (7%)
*Citrokcter feoseri*	3 (3%)
*Staphylococcus auricularis*	1 (1%)
*Pseudomonas putida*	1 (1%)
*Staphylococcus lentus*	5 (5%)
*Staphylococcus lugdunensis*	1 (1%)
*Acinetokcterliffii*	3 (3%)

## 5 Conclusion

We found that a wide range of pathogens affect DM patients, *Staphylococcus haemolyticus* is the most prevalent. Considerable antimicrobial resistance was observed. Tigecycline had a wide sensitivity spectrum and was effective against most of the bacteria. It could be used as an empirical antibiotic. However, choosing antibiotics to treat UTIs should be controlled by the local resistance pattern.

## Data Availability

The data used to support the findings of this study are included in the article.

## Abbreviations

DM: Diabetes mellitus

WHO: World Health Organization

IDF: International Diabetes Federation

UTIs: Urinary tract infections

SPSS: Statistical Package for Social Sciences
